# The Application of Kernel Ridge Regression for the Improvement of a Sensing Interferometric System

**DOI:** 10.3390/s25051292

**Published:** 2025-02-20

**Authors:** Ana Dinora Guzman-Chavez, Everardo Vargas-Rodriguez

**Affiliations:** Departamento de Estudios Multidisciplinarios, Universidad de Guanajuato, Yuriria 38940, Mexico; ad.guzman@ugto.mx

**Keywords:** kernel ridge regression, kernel function, multilayer interferometric system, optical sensor, thermal properties, temperature

## Abstract

Sensors based on interferometric systems have been studied due to their wide range of advantages, such as high sensitivity. For these types of sensors, traditional methods, which generally depend on the linear sensitivity of one variable, have been used to determine the measurand parameter. Usually, these methods are only effective for short measurement ranges, which is one of the main limiting factors of these sensors. In this work, it is shown that Kernel Ridge Regression (KRR), which is a machine learning method, can be applied to improve the range of measurement of multilayer interferometric sensors. This method estimates the value of a response variable (temperature) based on a set of spectral features, which are transformed by means of kernel functions. Here, these features were the wavelength positions and maximum amplitudes of some peaks of the interference spectrum of the sensing system. To sustain the application of the method, four kernel functions were used to estimate the values of the response variable. Finally, the results show that by implementing KRR with a Gaussian kernel, the temperature could be estimated with a root-mean-square error of 0.094 °C for the measurement range from 4.5 to 50 °C, which indicates that it was widened by a factor of eight compared with traditional methods.

## 1. Introduction

There are different data analysis techniques to predict output values based on a set of different features, including machine learning and deep learning techniques. The application of these techniques does not have an exclusive disciplinary area, and a wide number of models can be found in almost all areas of knowledge. Regarding optical sensors, they have been used to improve several detection capabilities [1,2,3,4]. For example, Maryamsadat et al. reported on non-invasive glucose monitoring [1], in which five different prediction models were applied, which were based on classification and regression methods, such as decision trees and artificial neural networks. In that work, the features used in the mathematical models were the transmission intensity of four wavelengths, and the estimated variable was the glucose concentration. In addition, Karapanagotis et al. proposed applying linear regression to estimate humidity and temperature from the output data of an optical sensor. That algorithm was trained by using Brillouin frequency shifts and the line widths of the fiber’s multipeak Brillouin spectrum as features and allowed the authors to minimize cross-sensitivity effects.

Specifically, machine learning and deep learning techniques have been used to simultaneously estimate two variables with high precision within a wide measurement range by analyzing interferometric optical sensor signals [5,6,7], which is difficult to achieve by applying conventional methods, such as a sensitivity matrix [8,9]. With this last method, the measurement ranges of the output variables are limited due to the cross-sensitivity between the independent variables (measurands). Furthermore, some machine learning methods have also been used to enlarge the measurement range of one output variable [6,10,11,12], which, typically, is limited due to the 2π ambiguity presented by interferometric optical sensors, which, usually, is related to the free spectral range of the interferometer (*FSR*). In another interesting example, the multiple regression model was implemented for the simultaneous measurement of the refractive index and temperature and to widen the measurement range by breaking the free spectral range limit [7]. In that work, different link functions were tested, and the considered features were obtained from the spectral patterns of the interferometric arrangement. Another example is the work by Zizheng Yue [10], in which a standard long short-term memory network was used to establish the relationship between the spectral intensity distribution information, sampled by an array waveguide output grating power data and the target measurand (displacement). According to the authors, the comparison between the real data and the estimated data reached a coefficient of determination of 0.99 in a wide measurement range.

In this work, it is shown that by applying Kernel Ridge Regression (KRR), it is possible to improve the measurement range of a multilayer interferometric sensing system. This method is based on a kernel function for which the inputs are two feature vectors that are extracted from the reflective spectrum of the interferometric system. Moreover, four kernel functions—Gaussian, exponential, Bessel, and inverse multi-quadratic functions—were used to estimate the values of the response variable (temperature) over a large measurement range. Here, it is shown that from a reduced experimental dataset, a larger synthetic dataset could be built to train and validate the model. Moreover, the synthetical dataset was divided into the training and evaluation datasets and the experimental dataset containing the original measured information. Furthermore, the efficiency of the model was evaluated with the root-mean-square error (*RMSE*) obtained for the three datasets. Here, the optimal parameters of the models were determined by considering these three *RMSE* values. Finally, it is presented that by implementing the algorithm with a Gaussian kernel, the temperature could be estimated with an *RMSE* of 0.094 °C in the experimental dataset, for a measurement range that covered eight *FSR* periods. This is quite important because, with traditional methods, the measurement range is usually limited to one *FSR* period.

## 2. Experimental Setup and Interferometric System Model

**Figure 1:**
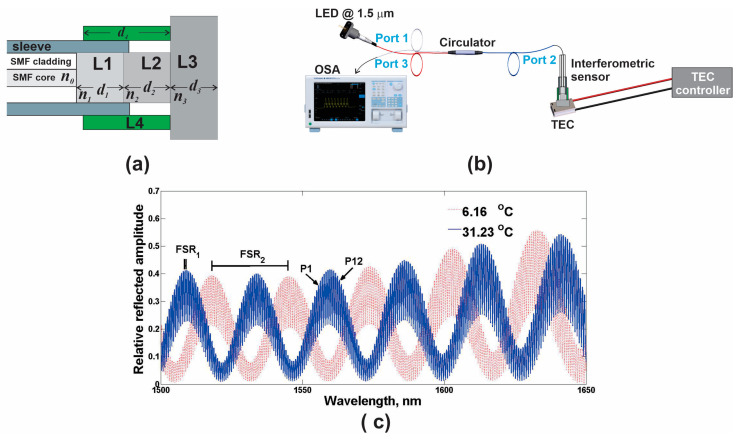
(**a**) Model of multilayer optical sensor with external expander, (**b**) schematic of experimental setup used to characterize interferometric sensor, and (**c**) example of recorded experimental spectra of interferometric sensor.

The physical model of the interferometric system used to study the viability of KRR to estimate the output variable is shown in Figure 1a. This system was based on an arrangement of three stacked layers (L1, L2, and L3) at the tip of a single-mode fiber (SMF) and an external expander (L4). Here, it is important to mention that the light was not in contact with layer L4. The details of the fabrication of the interferometric system and its mathematical model have previously been explained in detail in [13]. In addition, with the setup presented in Figure 1b, a set of experimental reflected spectra was obtained at different temperatures. Here, the light from the broadband source was transmitted to the fiber-coupled interferometric system through an optical circulator model 6015-3 (Thorlabs Inc., Newton, NJ, USA). The output spectrum of the interferometric system was monitored by an optical spectrum analyzer (OSA) (Yokogawa Test & Measurement Corporation, Musashino, Japan). Finally, a thermal electrical cooler (TEC) model HLD001 (Thorlabs Inc., Newton, NJ, USA) was used to control the temperature.

The relative reflected intensity of spectra can be modeled by using a mathematical model that considers the main reflected rays between layers [13]. The spectrum generated by one layer is a pattern of periodic fringes with an FSR that is inversely proportional to the thickness ($d_{i}$) and refractive index ($n_{i}$) of the layer. For a multilayer filter, the resulting fringe pattern is formed by the superposition of the patterns generated by each one of the layers. In this sense, if these patterns have different FSRs, the overall spectrum will be a pattern of fringes with modulated amplitudes. For our filter, the values of the thicknesses were $d_{1}$ = 321.9 nm, $d_{02}$ = 31.499 μm, $d_{03}$ = 495.38 μm, and $d_{04}$ = 4000 μm, and the values of the refractive indexes were $n_{0}$ = 1.44, $n_{1}$ = 1.2, $n_{02}$ = 1.45, and $n_{03}=3.41696+0.138497/A+0.013924/A^{2}-2.09\times 10^{-5}\lambda^{2}+1.48\times 10^{-7}\lambda^{4}$, where *A* = $\lambda^{2}$ − 0.028 [14], and λ is the wavelength. Here, $n_{1}$, $n_{02}$, and $n_{03}$ were considered constant functions within the wavelength range of 1500 ≤ λ ≤ 1650 nm. A couple of experimentally recorded spectra of the filter are shown in Figure 1c. The narrowest separation between the fringes with an ${FSR}_{1}$ corresponds to layer L3, and the separation between the peaks of the envelope with an ${FSR}_{2}$ corresponds to layer L2 [6].

As the materials of layers L2, L3, and L4 had thermal properties, the interference spectrum was shifted when the temperature was varied. The changes in the spectra were mainly governed by the values of the thermo-expansion ($\gamma_{i}$) and thermo-optic ($\rho_{i}$) coefficients and the thicknesses of each layer. In this sense, the thickness and the refractive index as a function of temperature (T) of each layer were $d_{i}=d_{0i}[1+\gamma_{i}(T-T_{o})]$ and $n_{i}=[n_{0i}+\rho_{i}(T-T_{0})],$ where $d_{0i}$ and $n_{0i}$ are the thickness and the refractive index at the reference temperature ($T_{0}$). An example of these changes is the behavior of the maximum amplitude (MA) and the wavelength positions (WP) of the peaks of the interference spectrum as a function of temperature shown in Figure 1c. In this figure, it is observed that the fringe amplitudes of the blue spectrum with an ${FSR}_{1},some\;labeled\;as\;P1,\ldots ,P12,$ were modulated by the fringes of the spectrum with an ${FSR}_{2}$. When the temperature of the measurement system was changed, the spectrum was shifted, causing a shift in the wavelength and a change in the maximum amplitude of the fringe peaks of the spectrum with an ${FSR}_{1}$. The behavior of the spectral response of this interferometer has been explained in detail in a previous work [13]. Here, it is important to mention that the slope associated with $\gamma_{4}$ was different for temperatures greater than 30 °C. Therefore, the change in the thickness $d_{2}$ was not constant as the temperature increased [15].

**Figure 2:**
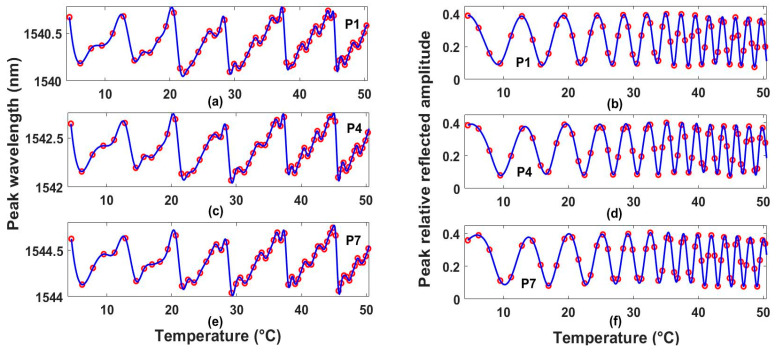
(**a**,**c**,**e**)Wavelength positions and (**b**,**d**,**f**) maximum amplitudes of peaks P1, P4, and P7 as a function of temperature, respectively. In these plots, circles are experimental data, while solid lines are interpolated data.

An example of the experimental behavior of the MA and the WP (red circles) of some peaks of the interference spectrum as a function of the temperature of the sensing interferometric system is shown in Figure 2. For this system, it can be seen that a quasi-linear relationship could be established between temperature and the MA of one fringe, but it was limited for a measurement range of shorter than one FSR, which was ~6 °C. For example, for P4, a linear relationship between the MA and the temperature could be defined for a measurement range from 9.3 to 12.9 °C (Figure 2d). Now, with respect to the linear relationship between temperature and the WP, the measurement range was also limited to less than one FSR. Here, it is shown that the KRR machine learning method could be able to estimate the response variable by considering a set of nonlinear explanatory variables. Here, firstly, an experimental dataset was formed with features extracted from all recorded reflection spectra. These features were the changes in the wavelength and maximum amplitude of some interference fringe peaks. Secondly, all features of the experimental dataset were interpolated to generate a synthetic dataset. By visual observation, it was expected that the synthetic dataset was reliable, since it fit very well all the experimental data points, as can be seen in Figure 2, where the blue lines represent the interpolated data.

## 3. Mathematical Model of KRR

In KRR, a set of features, $x_{n}\in X$, and the outcome values associated with these features $(y_{n}\in Y$) are used to estimate the value of a response variable $\left(\hat{y}\right)$ with the following expression:

(1) $$\hat{y}(x_{n})={Y^{T}\left(K+\varepsilon I\right)}^{-1}k(X,x_{n})$$

where ε>0 is a real regularization parameter, I is an N×N identity matrix, $x_{n}={\left[x_{1}\;x_{2}\;x_{3}\ldots x_{f}\right]}^{T}$ is a column vector that contains the features of the *n*-th case, f is the number of features, $X=[x_{1}\;x_{2}\;x_{3}\ldots x_{N}]$ is an f×N matrix, where N is the total number of cases, and $Y={\left[y_{1}\;y_{2}\;y_{3}\ldots y_{N}\right]}^{T}$ is a column vector. The matrix kernel, $K_{ij}=k\left(x_{i},x_{j}\right)$, is an N×N matrix, and it is expressed as follows:

(2) $$K=\left[\begin{array}{ccc} k\left(x_{1},x_{1}\right) & \cdots & k\left(x_{1},x_{N}\right)\\ \vdots & \ddots & \vdots \\ k\left(x_{N},x_{1}\right) & \cdots & k\left(x_{N},x_{N}\right) \end{array}\right]$$

Here, $k(X,x_{n})$ is a column vector, which is described by the following:

(3) $$k\left(X,x_{n}\right)={\left[k\left(x_{1},x_{n}\right)k\left(x_{2},x_{n}\right)k\left(x_{3},x_{n}\right)\ldots k\left(x_{N},x_{n}\right)\right]}^{T}$$

The four kernel functions that were applied for the data analysis of the sensing interferometric system are listed in Table 1. Moreover, for the evaluation of the goodness of estimation, the real (experimental) and estimated output values were compared with

(4) $$RMSE=\sqrt{{\sum_{n=1}^{N}\left(\hat{y}\left(x_{n}\right)-y_{n}\right)}^{2}/N}$$

### Implemented Algorithm

The KRR model is based on a parameter ε and a kernel function that has a parameter a. Now, to estimate the values of the response variable with high accuracy, an algorithm was implemented to find the optimal values of these parameters. In this algorithm, the heuristic method was used to explore all combinations formed with the proposed value sets (Table 1) to obtain the optimal a and ε parameters. The steps of this algorithm are as follows:

1. The data are divided into three sets: the training data, evaluation data, and experimental data.
2. The values of the f features for all the cases of the training data, the evaluation data, and the experimental data are put as the inputs of the matrixes $M_{t}$, $M_{e}$, and $M_{x}$, respectively. Their corresponding associated output values are the inputs of the vectors $Y_{t}$, $Y_{e}$, and $Y_{x}$, respectively.
3. A set of a values is proposed.
4. A value of a is chosen, and the matrix kernel $K_{t}$ is evaluated by using $M_{t}$ (Equation (2)).
5. A set of ε values is defined.
6. For one of the cases of the matrix $M_{t}$, its vector with features, $x_{t}$, and the a value is used to obtain the vector $k\left(M_{t},x_{t}\right)$ (Equation (3)). With the selected ε value and the vector $Y_{t}$, the value of the response variable $\hat{y_{t}}$ is estimated (Equation (1)). This step is repeated for all the cases.
7. The ${RMSE}^{t}$ is calculated (Equation (4)) with the values of $Y_{t}$ and $\hat{Y_{t}}$, where $\hat{Y_{t}}$ contains as inputs the values of $\hat{y_{t}}$. This step is repeated for all the values of ε.
8. The optimal value of ε is considered as ${(\varepsilon }_{f}),$ the one for which the minimum ${RMSE}^{t}$ is obtained.
9. For all the cases of the matrix $M_{e}$, the response variable $\left(\hat{y_{e}}\right)$ is estimated by using its features vector ($x_{e})$**,** the a and the $\varepsilon_{f}$ values, the matrix $K_{t}$, the vector $k\left(M_{t},x_{e}\right),$ and the vector $Y_{t}$.
10. The ${RMSE}^{e}$ is calculated with the values of $Y_{e}$ and $\hat{Y_{e}}$.
11. For all the cases of the matrix $M_{x}$ the response variable ($\hat{y_{x}})$ is estimated by using its features vector ($x_{x})$**,** the a and $\varepsilon_{f}$ values, the matrix $K_{t}$, the vector $k\left(M_{t},x_{x}\right),$ and the vector $Y_{t}$.
12. The ${RMSE}^{x}$ is calculated with the values of $Y_{x}$ and $\hat{Y_{x}}$.
13. Steps 4–12 are repeated for the entire set of values of a proposed in step 3.
14. The values of $\varepsilon^{KRR}$ and $a^{KRR}$ of the model are the values for which the values of ${RMSE}^{t}$, ${RMSE}^{e}$, and ${RMSE}^{x}$ present small values within the smaller range between these values. In this sense, these error values are labeled as ${RMSE}_{f}^{t}$, ${RMSE}_{f}^{e}$, and ${RMSE}_{f}^{x}$.
15. From the values obtained in step 14, the ${RMSE}_{f}^{x}$ value is considered as the RMSE value reached with the proposed algorithm, denoted as the ${RMSE}^{KRR}$.

## 4. Results

The experimental output data at different temperatures of the interferometric system were obtained by means of the implemented setup (Figure 1b). Here, the temperature was varied in the range from 4.5 to 50 °C in 97 steps. Moreover, for each temperature step, four spectra were recorded, and the time elapsed between the first and the fourth measured spectra was ~8 min. In this way, there were 388 experimentally recorded spectra, and from these, the MA and the WP of the first 12 peaks (P1–P12) of fringes occurring above 1540 nm were extracted, and these were taken as features (Figure 2). Later, these feature values were interpolated to strengthen the database. In this sense, a synthetic dataset of size 24 × 1000 was obtained. Afterward, the synthetic dataset was divided into the training dataset (TD) and the evaluation data (ED), for which 80% and 20% of the registers were randomly selected, respectively. Hence, the features of the TD were allocated in the matrix $M_{t}$ of size 24 × 800, while the features of the ED were in the matrix $M_{e}$ of size 24 × 200. Moreover, the synthetic values of the output variable for the TD and ED were allocated in the $Y_{t}$ and $Y_{e}$ vectors of size 800 × 1 and 200 × 1, respectively. Furthermore, the features of the experimental dataset (XD) were contained in the matrix $M_{x}$ of size 24 × 97, while the corresponding experimental outputs were saved in the $Y_{x}$ vector of size 97 × 1.

**Figure 3:**
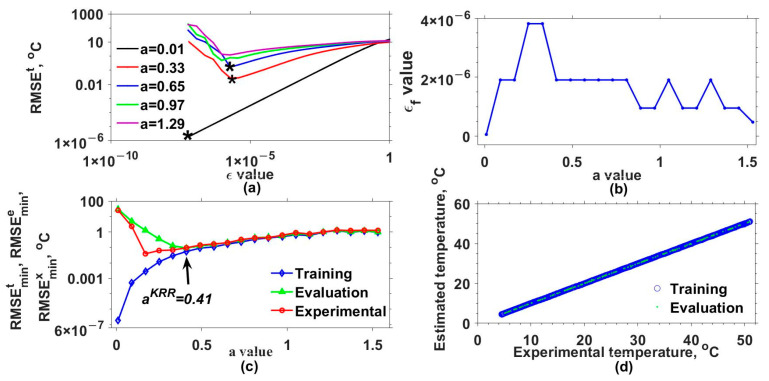
Results obtained with Gaussian kernel. (**a**) ${RMSE}^{t}$ values as a function of ε values for different a values, (**b**) $\varepsilon_{f}$ values as a function of a values, (**c**) ${RMSE}_{min}^{t}$, ${RMSE}_{min}^{e}$, and ${RMSE}_{min}^{x}$ values as a function of a values, and (**d**) estimated values as a function of real values of the response variable for the training data and test data. The minimum ${RMSE}^{t}$ values for three different cases are marked with asterisks.

**Figure 4:**
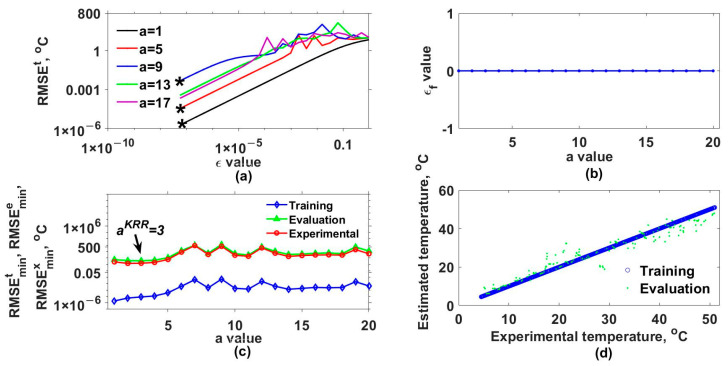
Results obtained with exponential kernel. (**a**) ${RMSE}^{t}$ values as a function of ε values for different a values, (**b**) $\varepsilon_{f}$ values as a function of a values, (**c**) ${RMSE}_{min}^{t}$, ${RMSE}_{min}^{e}$, and ${RMSE}_{min}^{x}$ values as a function of a values, and (**d**) estimated values as a function of real values of response variable for training data and test data. The minimum ${RMSE}^{t}$ values for three different cases are marked with asterisks.

**Figure 5:**
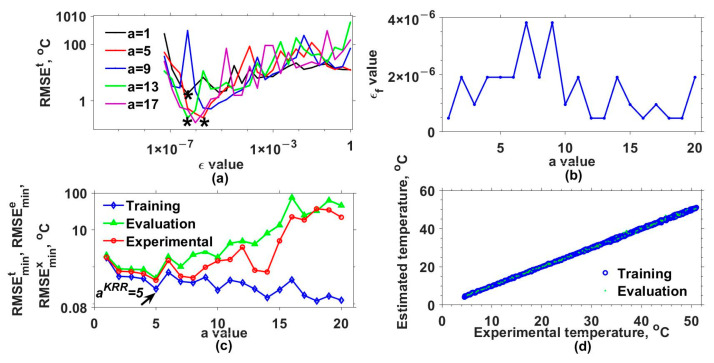
Results obtained with Bessel kernel. (**a**) ${RMSE}^{t}$ values as a function of ε values for different a values, (**b**) $\varepsilon_{f}$ values as a function of a values, (**c**) ${RMSE}_{min}^{t}$, ${RMSE}_{min}^{e}$, and ${RMSE}_{min}^{x}$ values as a function of a values, and (**d**) estimated values as a function of real values of response variable for training data and test data. The minimum ${RMSE}^{t}$ values for three different cases are marked with asterisks.

**Figure 6:**
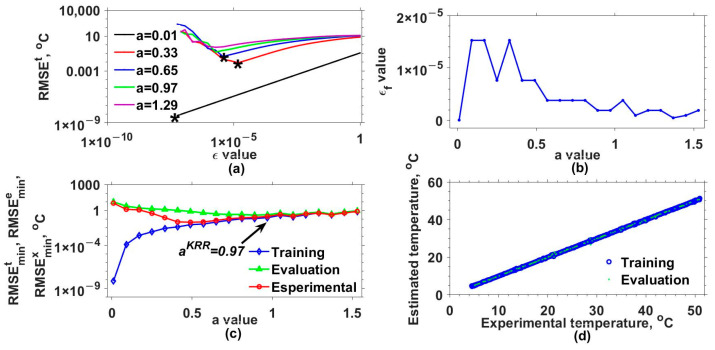
Results obtained with inverse multi-quadratic kernel. (**a**) ${RMSE}^{t}$ values as a function of ε values for different a values, (**b**) $\varepsilon_{f}$ values as a function of a values, (**c**) ${RMSE}_{min}^{t}$, ${RMSE}_{min}^{e}$, and ${RMSE}_{min}^{x}$ values as a function of a values, and (**d**) estimated values as a function of real values of response variable for training data and test data. The minimum ${RMSE}^{t}$ values for three different cases are marked with asterisks.

Later, the mathematical model for estimating the output of our interferometric system was implemented by considering different a and ε values and three kernel functions. Table 1 lists these functions and the a values used. Additionally, the set of ε values was defined as $\varepsilon_{i}=\varepsilon_{i-1}/2$ for i=2,3,..., 25 and $\varepsilon_{1}=1$. The results obtained with the Gaussian (GK), exponential (EK), Bessel (BK), and inverse multi-quadratic (MK) kernels are shown in Figure 3, Figure 4, Figure 5 and Figure 6, respectively. The ${RMSE}^{t}$ obtained for some of the used a values as a function of ε are shown in Figure 3a, Figure 4a, Figure 5a and Figure 6a for the GK, EK, BK, and MK, respectively. Here, it can be observed that for each a value, a curve was obtained. From each of these, the minimum ${RMSE}^{t}$ needed to be localized. For instance, in Figure 3a, the minimum ${RMSE}^{t}$ values for three different cases are marked with asterisks. In this way, for each a, the optimal ε was the one for which the smallest ${RMSE}^{t}$ was obtained, and we labeled it as $\varepsilon_{f}$. The obtained $\varepsilon_{f}$ values as a function of a are shown in Figure 3b, Figure 4b, Figure 5b and Figure 6b for each kernel.

**Figure 7:**
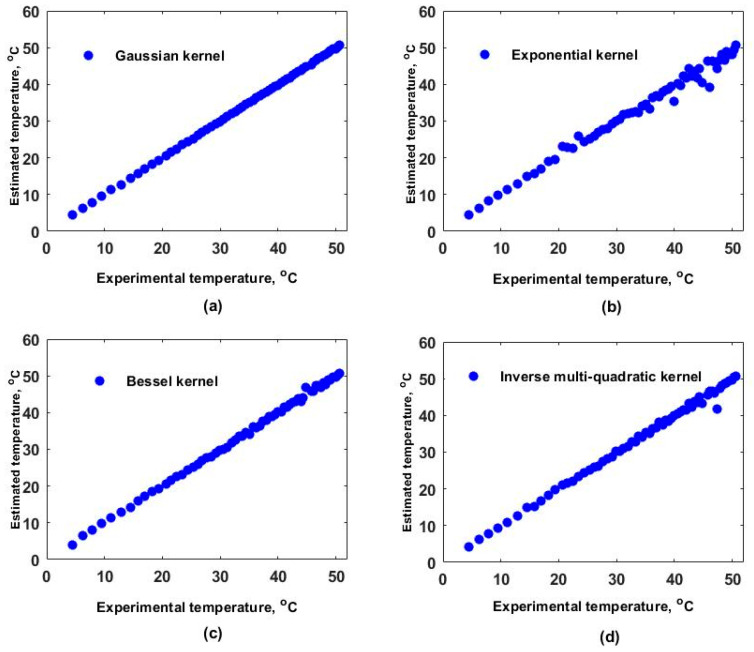
Estimated temperature values as a function of real experimental values. Results obtained with (**a**) Gaussian kernel, (**b**) exponential kernel, (**c**) Bessel kernel, and (**d**) inverse multi-quadratic kernel.

Furthermore, the model was evaluated again but now considering the optimal $\varepsilon_{f}$ and different a values, and the resulting RMSEs are shown in Figure 3c, Figure 4c, Figure 5c and Figure 6c for each kernel. In these figures, ${\mathrm{RMSE}}_{\min}^{t}$, ${\mathrm{RMSE}}_{\min}^{e}$, and ${\mathrm{RMSE}}_{\min}^{x}$ correspond to the TD, ED, and XD datasets. Here, the $a^{KRR}$ values were determined by considering the criteria mentioned in step 14 of algorithm 1. In this case, the smallest ranges and means between the ${\mathrm{RMSE}}_{\min}^{t}$, ${\mathrm{RMSE}}_{\min}^{e}$, ${\mathrm{and}\;\mathrm{RMSE}}_{\min}^{x}$ were 0.040–0.080 °C, 1.727–3.662 °C, 0.242–0.395 °C, and 0.173–0.225 °C for the GK, EK, BK, and MK, respectively. Now, with the selected $a^{KRR}$ and $\varepsilon^{KRR}$ values, the outputs of the synthetical TD (blue circles) and ED (cyan points) datasets were estimated ($\hat{y}$) and are shown as a function of the original synthetic (y) values in Figure 3d, Figure 4d, Figure 5d and Figure 6d for each kernel. In these figures, it can be observed that the RMSE values for the training dataset were fitted very well with all kernels; however, the GK provided the best fit for the evaluation (ED) dataset, while the poorest fit was obtained with the EK. Finally, by running the model considering the selected $a^{KRR}$ and $\varepsilon^{KRR}$ values, the estimated temperatures for the EX dataset were obtained, and these are plotted as a function of the experimental values in Figure 7. Here, it can be clearly observed that the best linear relationship between the estimated and experimental values was obtained when the GK was used.

The $a^{KRR}$, $\varepsilon^{KRR}$, ${RMSE}_{f}^{t}$, ${RMSE}_{f}^{e}$, and ${RMSE}_{f}^{x}$ values obtained by using the four kernel functions for the sensing interferometric systems are listed in Table 2. It can be seen that the best results were obtained with the Gaussian kernel for the measurement range used. Here, it should be pointed out that the KRR method allowed us to be able to estimate the temperature over a wide measurement range, which cannot be achieved by tracking only one variable, such as the maximum amplitude or the wavelength position of one fringe, as in the conventional method, due to the periodical behavior of the features (Figure 2). In addition, it should be noted that the implemented KRR model was trained with spectral features, which have physical restrictions governed by the behavior of the interferometer spectra. In our case, the physical constraints of each feature are listed in Table 3. Finally, it is important to mention that, in the way the model was trained, it was just validated for predicting the output variable (temperature) within the range from 4.5 to 50 °C, covering the experimental range for which the spectra were recorded. In future work, the capability of extending the predicting range outside the range for which the model was trained will be studied.

## 5. Conclusions

In this work, it was demonstrated that Kernel Ridge Regression (KRR) can be implemented to estimate the values of the response variable of interferometric sensing systems in a wide measurement range. The method is based on features that are transformed through kernel functions. Here, the temperature measured with a multilayer filter was estimated with high precision. Additionally, the features were extracted from its interference spectra. The method was applied considering four kernels (Gaussian, exponential, Bessel, and inverse multi-quadratic kernels). Here, it was shown that with the Gaussian kernel, the temperature could be estimated with a root-mean-square error of ~0.094 °C, within a measurement range of a ~45 °C width, covering a region of at least eight FSR periods, which demonstrates the robustness of the proposed method. This is quite important because with conventional methods, the estimation is usually valid for a period of one FSR.

## Figures and Tables

**Table 1:** Kernel functions used to estimate output data of sensing interferometric systems.

Kernel Name	Kernel Function	Parameter Grid Values Initial: Increment: Final
Gaussian	$k(x_{i},x_{j})=e^{-{\left(\left\|x_{i}-x_{j}\right\|\right)}^{2}}/2a^{2}$	a=0.01:0.08:1.53
Exponential	$k\left(x_{i},x_{j}\right)=e^{-{\left\|x_{i}-x_{j}\right\|}_{a}}$	a=1:1:20
Bessel	$k(x_{i},x_{j})=\frac{J_{2}(a*\left\|x_{i}-x_{j}\right\|)}{{\left\|x_{i}-x_{j}\right\|}^{-2}}$	a=1:1:20
Inverse multi-quadratic	$k(x_{i},x_{j})=\frac{1}{\sqrt{{\left\|x_{i}-x_{j}\right\|}^{2}+a^{2}}}$	a=0.01:0.08:1.53

**Table 2:** Results obtained by applying four kernel functions to output data of sensing interferometric system.

Kernel Name	$a^{KRR}$	$\varepsilon^{KRR}$	${RMSE}_{f}^{t}$ (°C)	${RMSE}_{f}^{e}$ (°C)	${RMSE}_{f}^{x}={RMSE}^{KRR}$ (°C)
Gaussian	0.41	$1.91\times 10^{-6}$	0.0543	0.092	0.094
Exponential	3	$5.96\times 10^{-8}$	$8.36\times 10^{-6}$	3.662	1.521
Bessel	5	$1.91\times 10^{-6}$	0.2534	0.4956	0.436
Inverse multi-quadratic	0.97	$1.91\times 10^{-6}$	0.1474	0.3210	0.209

**Table 3:** Physical constraint limits for each input feature of the KRR model.

	**Feature**						
	**WP1 (nm)**	**WP2 (nm)**	**WP3 (nm)**	**WP4 (nm)**	**WP5 (nm)**	**WP6 (nm)**	**WP7 (nm)**
Physical constraint limits	1540.00–1540.78	1540.71–1541.45	1541.37–1542.12	1542.02–1542.78	1542.67–1543.46	1543.32–1544.14	1543.97–1544.81
	**WP8 (nm)**	**WP9 (nm)**	**WP10 (nm)**	**WP11 (nm)**	**WP12 (nm)**	**AP1–AP12**	
Physical constraint limits	1544.62–1545.02	1545.28–1546.17	1545.93–1546.84	1546.59–1547.52	1547.25–1548.18	0.00–0.40	

## Data Availability

The raw data supporting the conclusions of this article will be made available by the authors upon request.
